# Supplementary material for: A Distinct Microglial Cell Population Expressing Both CD86 and CD206 Constitutes a Dominant Type and Executes Phagocytosis in Two Mouse Models of Retinal Degeneration
Source: Int J Mol Sci. 2023 Sep 18;24(18):14236. doi: 10.3390/ijms241814236 (PMC10532260; doi:10.3390/ijms241814236)
Supplement: Supplementary file 1 [file ijms-24-14236-s001.zip › ijms-2542079-supplementary.pdf]

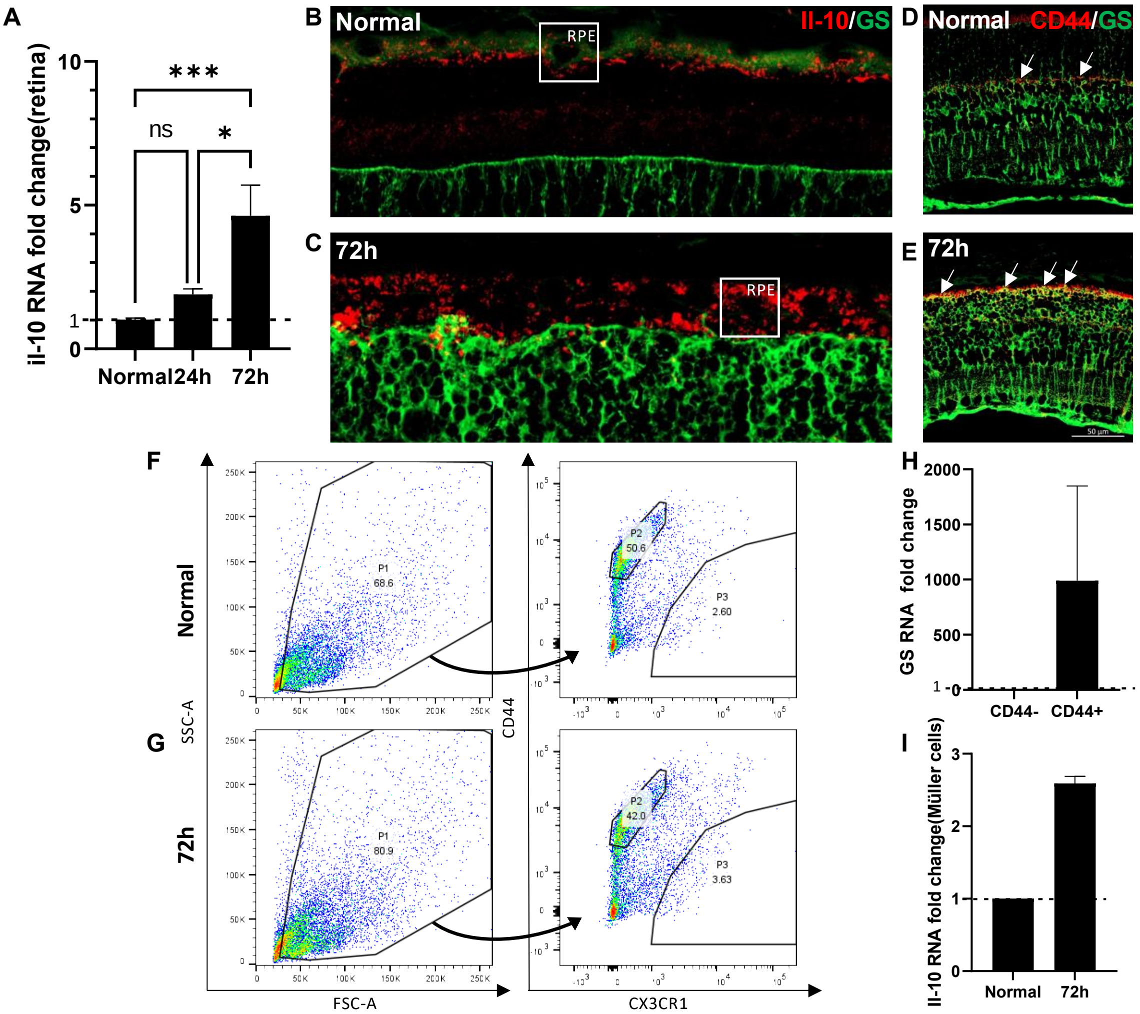

Figure S1. IL-10 and GS expression in the normal and blue LED-exposed Balb/c mice retina. (**A**) The RNA level of *Il-10* in whole retina increased at 72 h after LED exposure. (**B**,**C**) IL-10 was increased in the RPE after blue LED-induced RD retina 72 h (**C**, White square). (**D**,**E**) CD44 expression was increased in the end-feet of GS-labeled Müller glial cells at 72 h (**D**,**E**, white arrows). FACS was used to sort CD44 (+) and CX3CR1 (+) cells in the normal and blue LED-exposed Balb/c mice sclera and retinas (**F**,**G**). RNA level of the GS in FACS-sorted CX3CR1 cell population compared with FACS-sorted CD44 cell population (**H**). The RNA level of GS in CD44 positive cells is much higher than in CX3CR1 positive cells. RNA level of the *Il-10* was up-regulated in the CD44 sorted Müller glial cells from RD retina than normal retina (**I**). Data are presented by the mean ± S.E.M. (n = 7). *** $p < 0.05$, one-way ANOVA with Tukey's multiple comparison post-hoc test, * $p < 0.05$, Tukey's multiple comparison test. IL-10, interleukin 10; GS, glutamine synthetase.

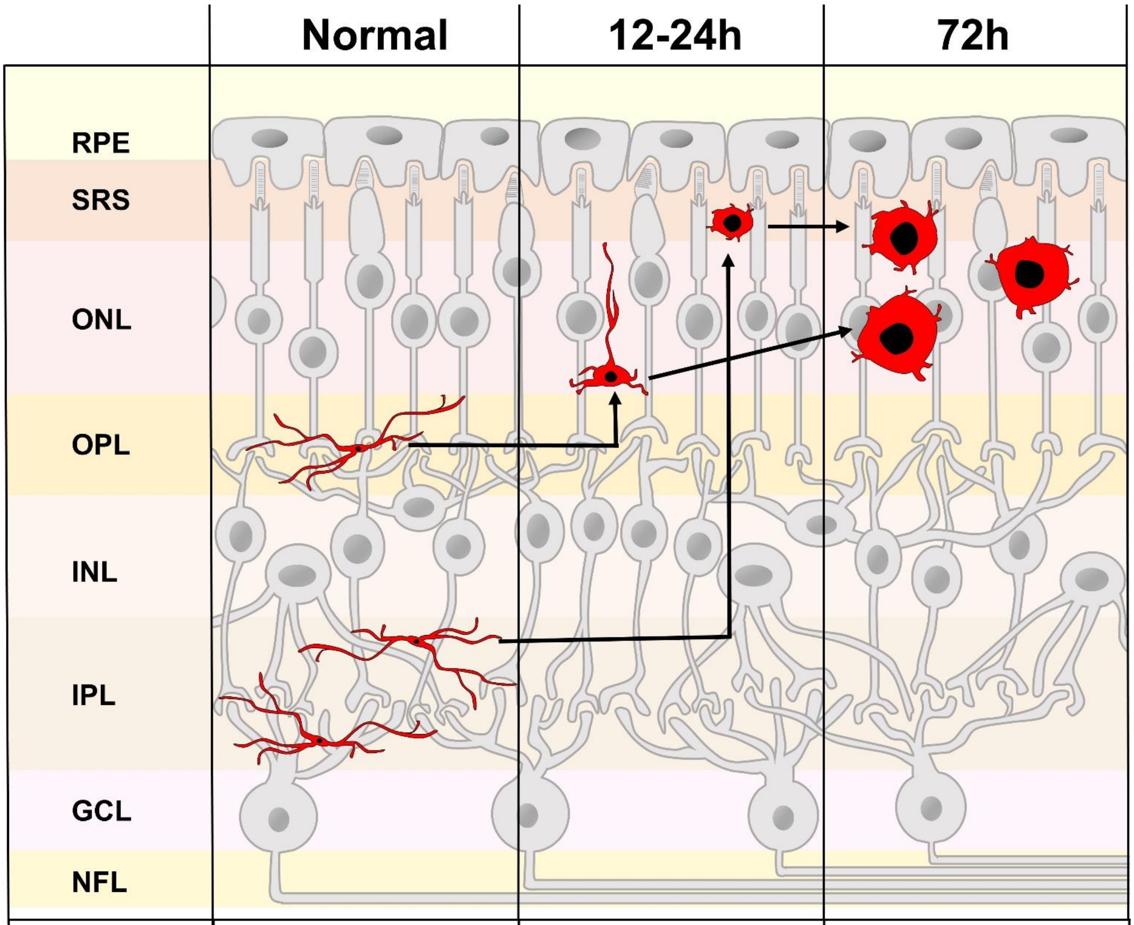

	Normal	12-24h	72h
P2RY12	○	○	×
CD86	×	○	○
CD206	×	×	○

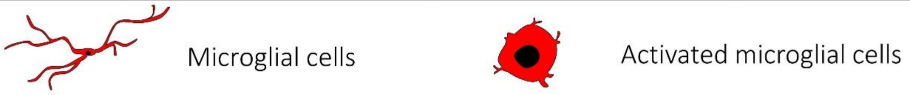

Figure S2. Graphical abstract depicting the alterations in the spatial and temporal profiles of microglia during the early stages of retinal degeneration in two mouse models. NFL, nerve fiber layer; GCL, ganglion cell layer; INL, inner nuclear layer; IPL, inner plexiform layer; ONL, outer nuclear layer; OPL, outer plexiform layer; RPE, retinal pigment epithelium; SRS, subretinal space.
